# From a Molecule to a Drug: Chemical Features Enhancing Pharmacological Potential II

**DOI:** 10.3390/molecules31040627

**Published:** 2026-02-12

**Authors:** Giovanni Ribaudo, Laura Orian

**Affiliations:** 1 Department of Molecular and Translational Medicine, Università di Brescia, Viale Europa 11, 25123 Brescia, Italy; 2 Dipartimento di Scienze Chimiche, Università degli Studi di Padova, Via Marzolo 1, 35131 Padova, Italy

The journey from a small molecule to an effective therapeutic agent is undoubtedly a complex and multidisciplinary challenge. Advances in chemistry, molecular biology, computational modeling, pharmacology, and materials science continuously reshape our understanding of how molecular features translate into pharmacological performance. The aim of this Special Issue, From a Molecule to a Drug: Chemical Features Enhancing Pharmacological Potential II, built on the success of the first volume, is to further explore the chemical principles that govern drug action and to showcase recent developments that bridge molecular design with therapeutic function. Drug discovery is no longer based on empirical screening but relies on rational and predictive design strategies. Today, researchers can interrogate molecular behavior, connecting structural features with biological responses, pharmacokinetic properties, and toxicological profiles. Nevertheless, the translation from promising compounds to clinically viable drugs is limited by constraints such as solubility, selectivity, metabolic stability, permeability, and toxicity. These aspects require a deep understanding of the chemical determinants underlying pharmacological success. One of the most straightforward approaches in modern drug discovery is drug repurposing [1,2,3], which involves identifying new therapeutic indications for existing, approved drugs, allowing savings in both time and costs. Additionally, minor modifications to the molecular structure of known drugs can be introduced to develop multifunctional or more potent drugs [4,5]; however, it is important to recognize that even minor modifications make it an entirely new drug. Another emerging direction is the integration of computational approaches with experimental design. Machine learning methods, molecular simulations, and quantum chemical calculations are increasingly pivotal in supporting decision-making in early-stage drug discovery, enabling faster identification of promising candidates while reducing the reliance on resource-intensive experimental cycles [6,7]. Such approaches demonstrate how modern chemistry now operates at the interface between theory and application, accelerating the path from concept to candidate.

This Special Issue gathers seven contributions published between 2022 and 2025, comprising six original research papers and a review article. More precisely, the research group of Mahmood presented 2-(4-isobutylphenyl)propanoic acid derivatives as antiproliferative agents based on a combination of computational and experimental data. Martínez-Campos and colleagues contributed a report on the microwave-assisted synthesis of antifungal compounds, while Liu and colleagues published a paper on dual-target inhibitors identified via innovative computational approaches. The team led by Mitrakas synthesized and tested 2-amino-*N*′-aroyl(het)arylhydrazides with antiproliferative activity, and Hong and colleagues reported the biological activity of rationally designed semi-synthetic derivatives. Eventually, Li and colleagues focused on innovative techniques in metabolite profiling. Lastly, Fabre and colleagues provided an overview of the current therapeutic options for age-related macular degeneration. A list of these contributions is reported below, following the order described in the paragraph above:

- Mahmood, S.; Khan, S.; Rasul, A.; Christensen, J.; Abourehab, M. Ultrasound Assisted Synthesis and In Silico Modelling of 1,2,4-Triazole Coupled Acetamide Derivatives of 2-(4-Isobutylphenyl)propanoic acid as Potential Anticancer Agents. *Molecules* **2022**, *27*(22), 7984. https://doi.org/10.3390/molecules27227984.
- Martínez-Campos, Z.; Elizondo-Zertuche, M.; Hernández-Núñez, E.; Hernández-Fernández, E.; Robledo-Leal, E.; López-Cortina, S. Microwave-Assisted Synthesis of Aminophosphonic Derivatives and Their Antifungal Evaluation against *Lomentospora prolificans*. *Molecules* **2023**, *28*(10), 3995. https://doi.org/10.3390/molecules28103995.
- Liu, L.; Na, R.; Yang, L.; Liu, J.; Tan, Y.; Zhao, X.; Huang, X.; Chen, X. A Workflow Combining Machine Learning with Molecular Simulations Uncovers Potential Dual-Target Inhibitors against BTK and JAK3. *Molecules* **2023**, *28*(20), 7140. https://doi.org/10.3390/molecules28207140.
- Mitrakas, A.; Stathopoulou, M.; Mikra, C.; Konstantinou, C.; Rizos, S.; Malichetoudi, S.; Koumbis, A.; Koffa, M.; Fylaktakidou, K. Synthesis of 2-Amino-*N*′-aroyl(het)arylhydrazides, DNA Photocleavage, Molecular Docking and Cytotoxicity Studies against Melanoma CarB Cell Lines. *Molecules* **2024**, *29*(3), 647. https://doi.org/10.3390/molecules29030647.
- Hong, S.; Baravkar, S.; Lu, Y.; Masoud, A.; Zhao, Q.; Zhou, W. Molecular Modification of Queen Bee Acid and 10-Hydroxydecanoic Acid with Specific Tripeptides: Rational Design, Organic Synthesis, and Assessment for Prohealing and Antimicrobial Hydrogel Properties. *Molecules* **2025**, *30*(3), 615. https://doi.org/10.3390/molecules30030615.
- Li, X.; Zhang, Q.; Li, Y.; Qin, L.; Wu, D.; Tan, D.; Xie, J.; Wu, J.; Yang, Q.; Lu, Y.; Zhao, Y.; Fan, Q.; Wu, X.; He, Y. Utilizing High-Resolution Mass Spectrometry Data Mining Strategy in R Programming Language for Rapid Annotation of Absorbed Prototypes and Metabolites of Gypenosides. *Molecules* **2025**, *30*(4), 779. https://doi.org/10.3390/molecules30040779.
- Fabre, M.; Mateo, L.; Lamaa, D.; Baillif, S.; Pagès, G.; Demange, L.; Ronco, C.; Benhida, R. Recent Advances in Age-Related Macular Degeneration Therapies. *Molecules* **2022**, *27*(16), 5089. https://doi.org/10.3390/molecules27165089.

We hope that this collection provides a snapshot of current progress and, most of all, inspiration for future research. The diversity of approaches presented here highlights the central role of chemical sciences in advancing pharmacology. Moreover, it is important to acknowledge that this volume had an international reach, as authors from all over the world submitted their contributions.

We are grateful to all authors who contributed their high-quality work to this Special Issue, as well as to the reviewers for their careful evaluations and constructive feedback. We also thank the editorial staff for their continuous support throughout the preparation of this issue.
